# Ca^2+^ Promoted the Low Transformation Efficiency of Plasmid DNA Exposed to PAH Contaminants

**DOI:** 10.1371/journal.pone.0058238

**Published:** 2013-03-06

**Authors:** Fuxing Kang, Hong Wang, Yanzheng Gao, Jian Long, Qian Wang

**Affiliations:** 1 Institute of Organic Contaminant Control and Soil Remediation, College of Resource and Environmental Sciences, Nanjing Agricultural University, Nanjing, People’s Republic of China; 2 Key Laboratory for Information System of Mountainous Areas and Protection of Ecological Environment of Guizhou Province, Guizhou Normal University, Guiyang, People’s Republic of China; 3 School of Earth Science and Engineering, Nanjing University, Nanjing, People’s Republic of China; Shantou University Medical College, China

## Abstract

The effects of interactions between genetic materials and polycyclic aromatic hydrocarbons (PAHs) on gene expression in the extracellular environment remain to be elucidated and little information is currently available on the effect of ionic strength on the transformation of plasmid DNA exposed to PAHs. Phenanthrene and pyrene were used as representative PAHs to evaluate the transformation of plasmid DNA after PAH exposure and to determine the role of Ca^2+^ during the transformation. Plasmid DNA exposed to the test PAHs demonstrated low transformation efficiency. In the absence of PAHs, the transformation efficiency was 4.7 log units; however, the efficiency decreased to 3.72–3.14 log units with phenanthrene/pyrene exposures of 50 µg·L^–1^. The addition of Ca^2+^ enhanced the low transformation efficiency of DNA exposed to PAHs. Based on the co-sorption of Ca^2+^ and phenanthrene/pyrene by DNA, we employed Fourier-transform infrared spectroscopy (FTIR), X-ray photoelectron spectroscopy (XPS), and mass spectrometry (MS) to determine the mechanisms involved in PAH-induced DNA transformation. The observed low transformation efficiency of DNA exposed to either phenanthrene or pyrene can be attributed to a broken hydrogen bond in the double helix caused by planar PAHs. Added Ca^2+^ formed strong electrovalent bonds with “–POO^–^–” groups in the DNA, weakening the interaction between PAHs and DNA based on weak molecular forces. This decreased the damage of PAHs to hydrogen bonds in double-stranded DNA by isolating DNA molecules from PAHs and consequently enhanced the transformation efficiency of DNA exposed to PAH contaminants. The findings provide insight into the effects of anthropogenic trace PAHs on DNA transfer in natural environments.

## Introduction

Genetic transformation is a process in which a bacterial recipient takes up exogenous free DNA and incorporates it into its own chromosome by homologous recombination or converts it into an autonomous extrachromosomal replicon [1]. Genetic transformation plays an important role in biological heredity and variation, ecological and genetic diversity, and biological evolution [2]–[4]. Upon the death of an organism, intracellular germplasm accompanied by other extracellular materials is released into soil and water environments, transferred to other biological cells, and expressed in the new host. Such horizontal gene transfers (HGTs) among biological species have been widely reported. For example, up to 10–16% of *Escherichia coli* DNA was acquired through HGT [3], [4]. Additionally, *E. coli* isolated from the intestine of Japanese patients contained gene segments that originated from the oceanic environment by way of edible seafood [5], [6], which indicates that gene transfer between species is ubiquitous in natural environments [7]–[9].

HGTs may be affected by organic contaminants, including polycyclic aromatic hydrocarbons (PAHs), which are considered lipophilic persistent organic contaminants; they are by-products of the incomplete combustion or pyrolysis of organic materials and the main pollutants of concern in the environment due to their persistence and strong mutagenic/carcinogenic properties [10]–[12]. With increasing discharge into the atmosphere, soil, and water columns through various pathways, PAHs pose great threats to human health and may alter some natural processes. For example, enzymatic interactions between PAHs and intracellular DNA induce changes in genetic information via mutation, teratogenesis, and carcinogenesis [13]. These effects are mainly based on enzymatic DNA adducts resulting from the direct combination of PAHs with bases of intracellular DNA [14]–[16]. Such processes can alter genetic information and even induce cell suicide. However, the effects of interactions between genetic materials and PAHs on gene expression in extracellular environments remain unknown.

In nature, Ca^2+^ is a ubiquitous ion in water and soil environments. Furthermore, the effect of the interaction between genetic materials and PAHs on genetic expression in extracellular environments may be affected by the ionic strength of that environment. Many studies have documented the effect of ionic strength on DNA transformations, suggesting that Ca^2+^/Mg^2+^ at concentrations of more than 80 mmol·L^–1^ have a positive effect on DNA transformation [17]. This process is explained by the formation of hydroxyl-calcium phosphate that protects DNA from the enzymatic degradation of the cell surface and assists the plasmid in penetrating the host cell [1], [17]. Despite this, few reports related to the effect of ionic strength on the transformation of plasmid DNA exposed to PAHs have been published.

Thus, the aims of this study were to evaluate the effect of PAH exposure on DNA transformation and the effect of Ca^2+^ on the transformation of plasmid DNA exposed to PAHs. The results will strengthen our understanding of how anthropogenic trace PAHs affect biological heredity and variation, ecological and genetic diversity, and biological evolution in natural environments.

## Materials and Methods

### Chemicals

Phenanthrene and pyrene, which were used as representative PAHs (purity of >98%), were purchased from Aldrich Chemical Co. Ltd. The molecular weights (*M*_w_s), solubility in water at 25°C (*S*_w_), and log *K*_ow_ (*K*_ow_ denotes the octanol–water partition coefficient) of phenanthrene and pyrene are 178 and 202 g·mol^–1^, 1.18 and 0.12 mg·L^–1^, and 4.46 and 4.88, respectively [10], [18]–[20]. Two DNA molecules were used throughout the entire experiment. A pUC19 plasmid DNA (with a gene insert encoding ampicillin resistance) that achieves HGT was used to investigate the transformation of DNA exposed to both phenanthrene/pyrene and Ca^2+^. Additionally, the underlying mechanism of transformation was investigated based on Ca^2+^-controlled PAH adsorption and the results of Fourier-transform infrared (FTIR), mass spectrometry (MS), and X-ray photoelectron spectroscopy (XPS). In the sorption experiment, salmon sperm DNA was used to supply the adequate quantities of DNA necessary to facilitate a Ca^2+^-controlled PAH adsorption test.

### Culture Media

Three different culture media were prepared. A Luria-Bertani (LB) liquid culture (tryptone, 10 g; yeast extract, 5 g; NaCl, 10 g, ultrapure water, 800 mL) was pH-adjusted to 8.0 using a dilute NaOH solution and diluted with ultrapure water to a volume of 1 L. An LB solid culture was prepared by adding agar powder (12 g) to the LB liquid media (pH 8.0, volume 1 L) and then heated to 90°C for 10 min to melt the agar powder. A super optimal broth with catabolite repression (SOC) liquid culture (tryptone, 20 g; yeast extract, 5 g; NaCl, 0.5 g; glucose, 4 g; ultrapure water, 1 L) was prepared, and all three media were sterilized in an autoclave at 120°C for 30 min.

### Bacterial Strain and Cell Preparation

An *E. coli DH5α* strain was chosen as the plasmid DNA acceptor. To clearly observe the plasmid DNA transformants, an accelerating transformation protocol was followed based on the protocol described by Lou et al. [17]. The *DH5α* strain was inoculated into the LB liquid culture (1% volume ratio) and incubated in the logarithmic growth phase (OD_600_ = 0.4–0.6) at 37°C. The bacteria-inoculated liquid was cooled in an ice-water bath for 10 min and centrifuged for 10 min at 3000 rpm at 4°C to obtain cell pellets, which were resuspended in 40 mL of a pre-cooled CaCl_2_ solution (0.05 mol·L^–1^). The cell suspension was then placed in an ice-water bath for 30 min and centrifuged for 10 min at 4°C. The supernatant was removed, and the cell pellets were resuspended in 40 mL of a pre-cooled glycerol–CaCl_2_ solution (CaCl_2_: 0.05 mol·L^–1^, glycerol: 15% v/v). The treated cells were carefully and gently suspended in an ice-water bath for 5 min to obtain the *E*. *coli DH5α*-competent cells. The competent cells (200 µL) were packaged in 10-mL Eppendorf tubes and stored at –70°C.

### Plasmid DNA Exposure to PAHs in the Presence of Ca^2+^

Prior to the experiment, 25 µg of plasmid DNA was dissolved in a Tris-HCl solution (10 mmol·L^–1^, 500 µL, pH 7.0) to prepare the plasmid DNA solution. A 100-µL aliquot of the Tris-HCl solution (10 mmol·L^–1^, pH 7.0) was added in advance to another 5-mL test tube. The plasmid DNA solution (5 µL) was injected into a test tube using an injector with a scale of 10 µL. In succession, the PAH and Ca^2+^ solutions were added to the test tube containing the diluted plasmid DNA solution to establish the desired Ca^2+^/PAH concentration gradients. In all reactive solutions the DNA concentrations were the same, but the Ca^2+^ and PAH concentration gradients differed. Finally, aluminum foil was used to envelop the mouth of test tube, and a rubber band was used to hold the film to prevent evaporation. The treated plasmid DNA solutions were prepared in triplicate, incubated at 25°C, and shaken at 180 rpm for 2 h.

### Transformation of Plasmid DNA

The 500 µL of competent cells was stored in an ultralow-temperature refrigerator and thawed on ice. The plasmid DNA solutions (5 µL) containing PAH/Ca^2+^ after incubation for 2 h were added to the competent cells. The mix of cells and plasmids was placed in an ice-water bath for 3 min and immediately heat-shocked at 42°C. Following heat shock for 90 s, the bacterial solutions of imbibing the DNA were immediately placed in an ice-water bath for 3 min. SOC (750 µL) was added to the cell solution and the mixture was shaken at 120 rpm for 45 min at 37°C to express the *pUC19* gene. The bacteria-inoculated liquid (100 µL) was uniformly spread on the surface of the LB solid culture media containing 100 mg·L^–1^ of ampicillin sodium. Concentrations of PAHs in plasmid DNA solutions were diluted into the 1/100 equivalent as original concentrations. Preliminary results confirmed that the concentration of phenanthrene/pyrene (ppb) had a negligible effect on the growth of *E. coli* cells.

The petri dish was left upright for 30 min to ensure that the *E. coli* medium was fully imbibed by the solid culture medium. The dish was then inverted for 36 h at 37°C. The transformation efficiency was defined as the ratio of the number of transformants (unit number) versus the mass of the added plasmid DNA (µg). This ratio was used to calculate the log_10_ (number of transformants·µg^–1^ DNA) exposed to trace PAHs according to the following formula [21]:
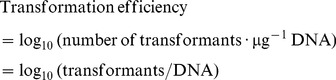
(1)


### PAH Adsorption onto DNA in the Presence of Ca^2+^

The methanol–water stock solutions of phenanthrene and pyrene (400 µg·L^–1^) were added to the DNA solution to obtain mixtures of PAH and DNA (methanol concentration was <0.00625%). The Ca^2+^ stock solution (0.4 mol·L^–1^) was added according to the desired Ca^2+^ concentration. The mixtures of Ca^2+^, DNA, and PAHs were incubated at 25°C for 24 h, and PAH concentrations of the solution were determined using high-performance liquid chromatography (HPLC).

### Analysis of PAHs

Previous studies have used similar methods for determining the amount of hydrophobic organic contaminants adsorbed by low-molecular-weight organic matter [22], [23]. To determine the free PAHs (i.e., those not absorbed by DNA), their concentration in aqueous solution was measured using a Shimadzu HPLC (LC-20AT) equipped with a fluorescence detector and a Ф4.6×150 mm reverse phase C18 column (Shimadzu®). The PAHs adsorbed by DNA could not be detected because DNA quenches PAH fluorescence [22], [24]. PAHs in solution and PAHs adsorbed by DNA were separated based on their retention times using the C18 column (Shimadzu).

A fluorescence detector was used to determine the concentrations of phenanthrene and pyrene in solution. The excitation wavelength for phenanthrene was set at 250 nm, and the emission wavelength was collected at 362 nm. The excitation and emission wavelengths for pyrene were 336 nm and 362 nm, respectively. The mobile phase was a spectrum of pure methanol (90%) and ultrapure water (10%) at a flow rate of 1.0 mL·min^–1^. Sample injection volume was 10 µL, and the column temperature was constant at 40°C.

### Detection of Potential DNA–PAH Adducts

To determine the inhibitory mechanism of phenanthrene and pyrene on DNA transformation, we detected potential adducts of DNA–PAHs using HPLC-MS. All samples of DNA, DNA–phenanthrene/pyrene, and DNA–phenanthrene/pyrene-Ca^2+^ were precipitated using pre-frozen ethanol, with a volume ratio of DNA solutions to ethanol of 1∶3. The precipitates from these solutions were separated by centrifugation at 3000 rpm and 4°C for 20 min. Supernatants were discarded, and the pellets were fully dried for 24 h by a vacuum freeze-dryer at –65°C (7670530; Labconco).

A modified method was applied to hydrolyze the DNA, DNA–phenanthrene/pyrene, and DNA–phenanthrene/pyrene-Ca^2+^ granules. Perchloric acid (10 µL, 75%) was added to 0.5 mg DNA in a glass tube, and the nozzles were fully sealed using a glass plug. The tubes were placed in a thermostatic water bath for 40 min at 100°C. After adding ultrapure water to the hydrolyte (1 mL), the solutions were separated for 10 min by centrifugation at 10,000 rpm and 4°C. The supernatant was collected and stored at –40°C for HPLC-MS analysis (Thermo Scientific). During hydrolysis, ionic compounds based on “Ca^2+^–DNA” could not be detected by HPLC-MS because the perchloric acid induced the breaking of ionic bonds (ionic compounds of Ca^2+^–DNA were analyzed using XPS).

The mobile phase for HPLC-MS analysis was a spectrum of pure methanol (50%) and ultrapure water (50%) with a flow rate of 1.0 mL·min^–1^ and a constant column temperature at 40°C. A reversed-phase column (Xterra MS-C8, 1.0 mm i.d.×100 mm, 3.5 µm; Waters) was used throughout the entire study. The sample injection volume was 10 µL. Positive ion mode electrospray ionization (ESI) was used to analyze the nucleotides.

### FTIR and XPS Analysis

The functional group structures of DNA, DNA–phenanthrene/pyrene, and DNA–phenanthrene/pyrene-Ca^2+^ were analyzed using FTIR. After mixing these samples with potassium bromide (Kbr) at a ratio of 1∶500, the mixtures were ground into fine granules with a carnelian mortar, and the disc-shaped samples were analyzed using a Nicolet FTIR spectrometer (Impact 420 model, Thermo Scientific) to determine their structures.

To determine the elemental forms of the resulting treated DNA, dried samples were analyzed by XPS using a PHI5000 VersaProbe (ULVAC-PHI®) with an AlKa X-ray source (1486.6 eV of photons) at 15 kV and 25 W. The data were recorded at room temperature and at a pressure below 6.7×10^–8 ^Pa. All binding energies were referenced to the neutral C1s peak at 285.0 eV to compensate for surface-charge effects.

### Data Analysis

**Figure 1:**
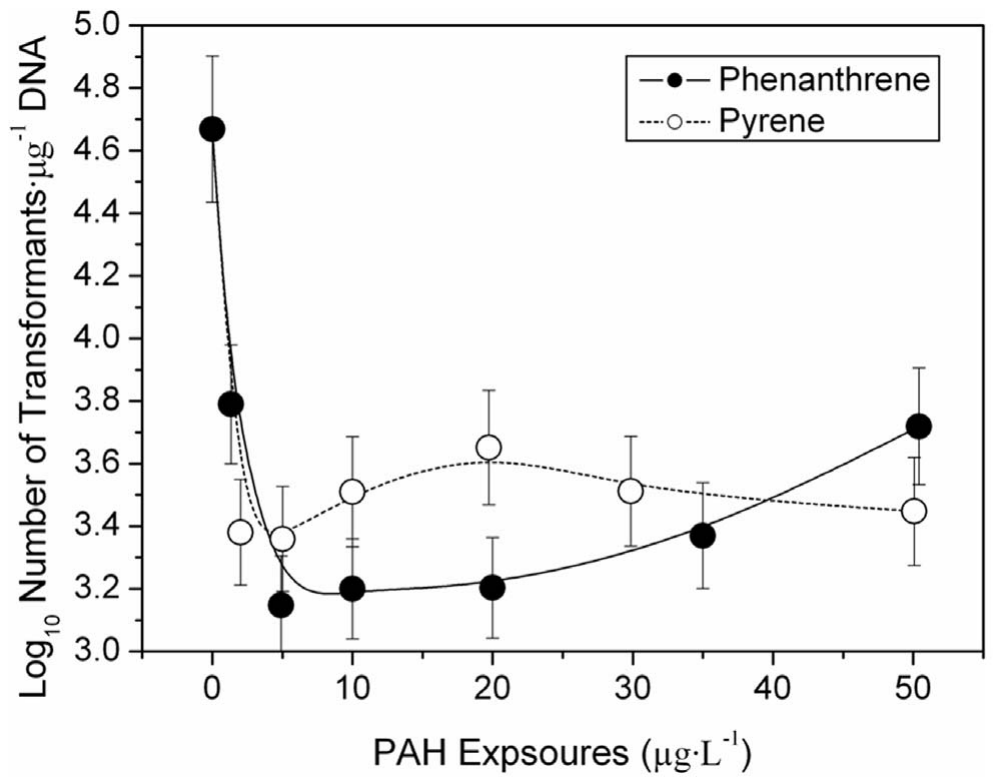
The transformation efficiency of plasmid DNA as a function of phenanthrene/pyrene concentrations. The transformation efficiency was calculated according to Equation I. Error bars represent 1 standard deviation (n = 3).

**Figure 2:**
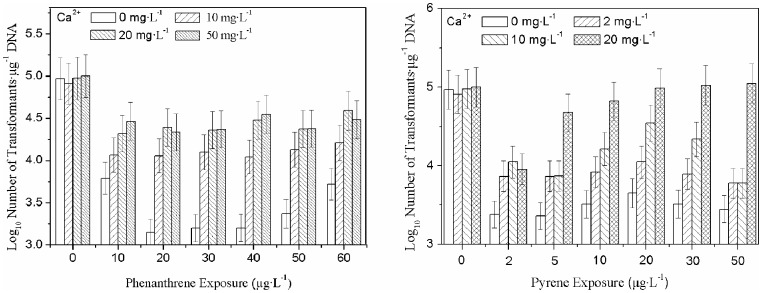
The Ca^2+^-influenced transformation efficiency of plasmid DNA exposed to phenanthrene (left) and pyrene (right). The transformation efficiency was calculated according to Equation I. Error bars represent 1 standard deviation (n = 3).

**Figure 3:**
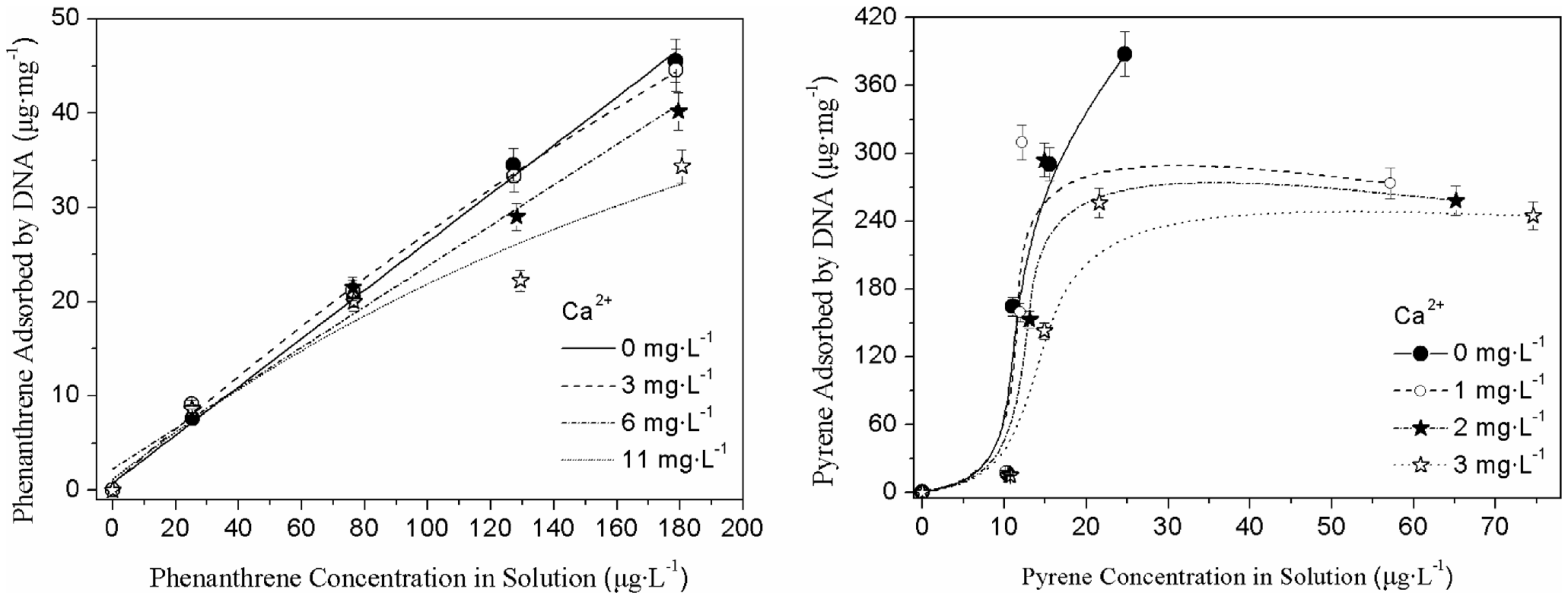
The influence of Ca^2+^ on phenanthrene (left) and pyrene (right) adsorption by DNA. The concentration of sorbent (DNA) in solution was 11.73 mg·L^–1^. Error bars represent 1 standard deviation (n = 3).

The data for adsorption and DNA transformation were analyzed using Origin software (version 8.0). The averaged values exhibited in Figures 1, 2, 3 were based on triplicate samples (n = 3), and the error bars represent 1 standard deviation. The HPLC-MS data were analyzed using Xcalibur software (version 1.4; Thermo Scientific).

## Results and Discussion

### Low Transformation Efficiency of Plasmid DNA Exposed to Trace PAHs

**Figure 4:**
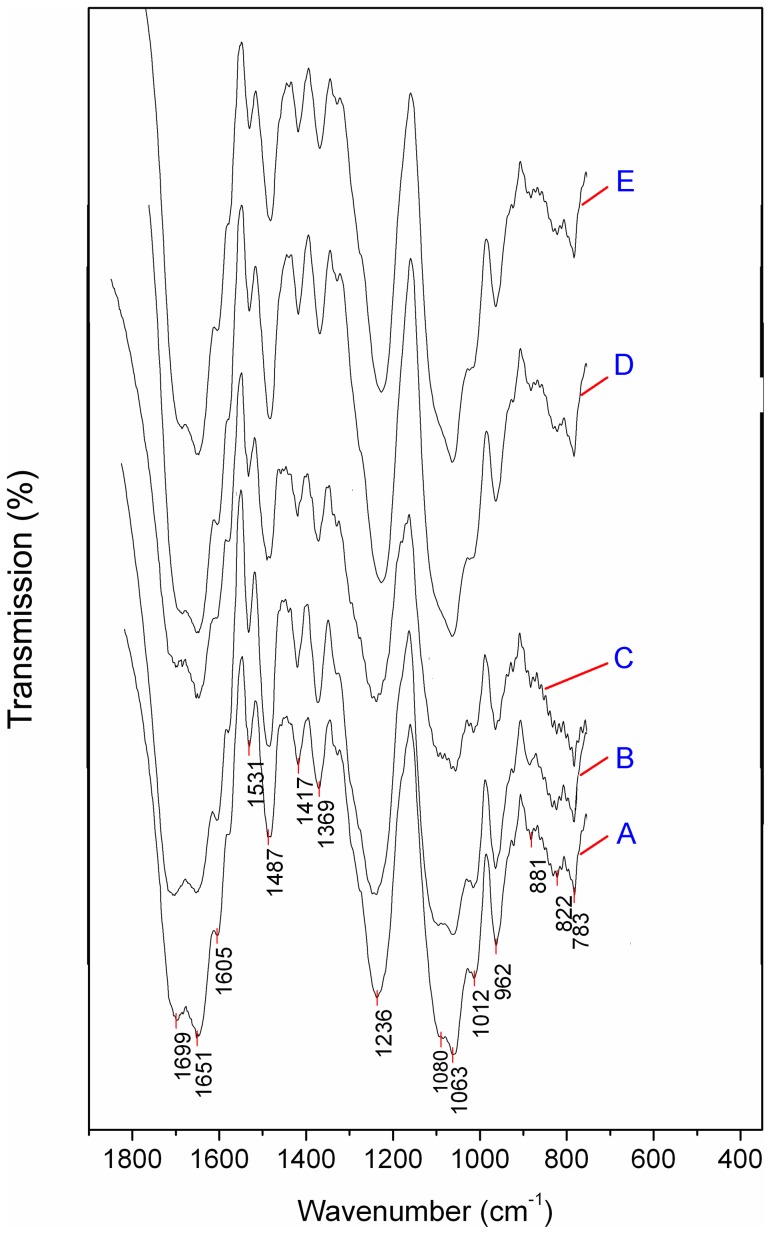
The FTIR spectra for DNA (A), DNA–phenanthrene (B), DNA–pyrene (C), DNA–phenanthrene-Ca (D), and DNA–pyrene-Ca (E). In (D) and (E), the peak at 1080 cm^–1^, which was related to the symmetrical stretch vibration of phosphate functional groups (PO_2_^–^–O–CH_2_), disappeared, indicating the production of hydroxy calcium phosphate by electrovalent bonds.

**Figure 5:**
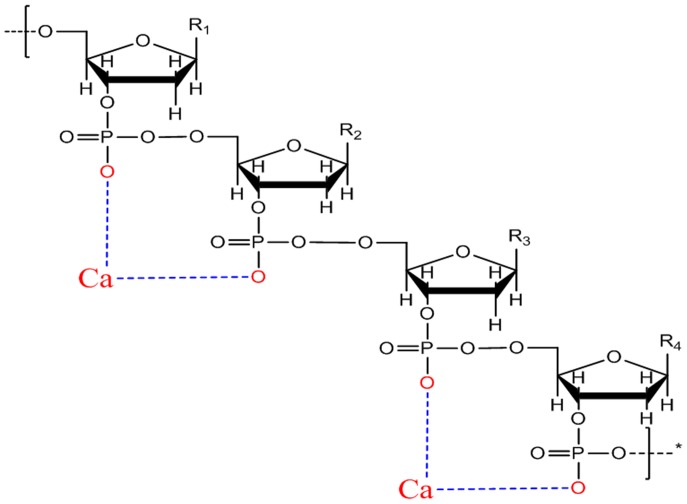
Molecular model combining the –POO^–^– group of DNA with Ca^2+^ linked by an electrovalent bond. R_1_, R_2_, R_3_, and R_4_ represent the different bases in DNA, and the blue dashed line represents the electrovalent bond between Ca^2+^ and the –POO^–^– group.

**Figure 6:**
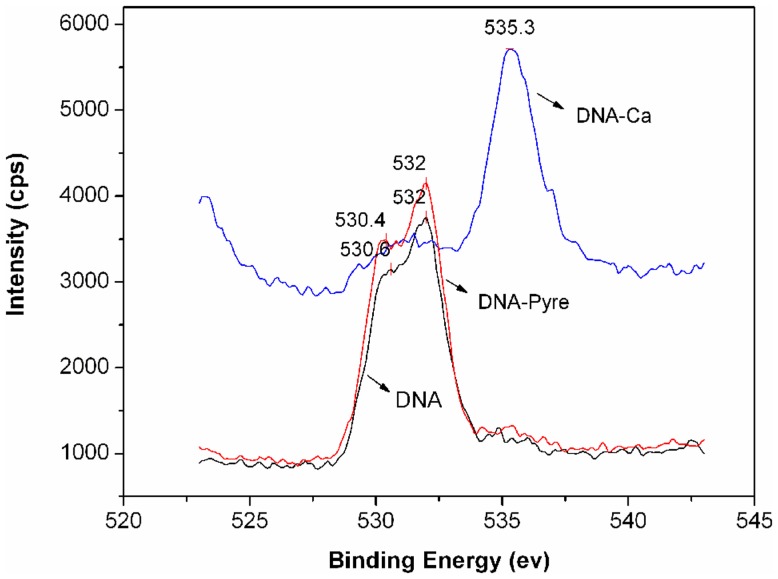
XPS analysis of oxygen in DNA, DNA–pyrene, and DNA–Ca.

**Figure 7:**
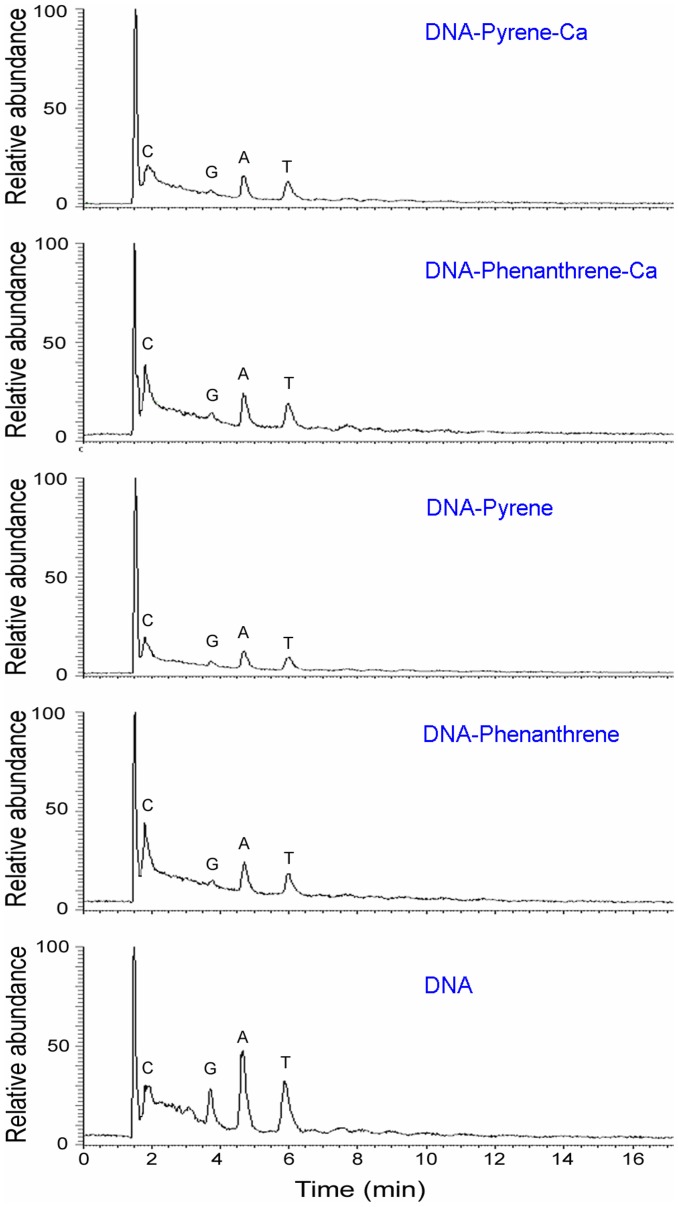
Chromatogram of the four bases of DNA, DNA–phenanthrene, DNA–phenanthrene-Ca^2+^, DNA–pyrene, and DNA–pyrene-Ca^2+^. C: cytosine, G: guanine, A: adenine, T: thymine. The HPLC-MS spectra showed an absence of PAH–DNA adducts.

**Figure 8:**
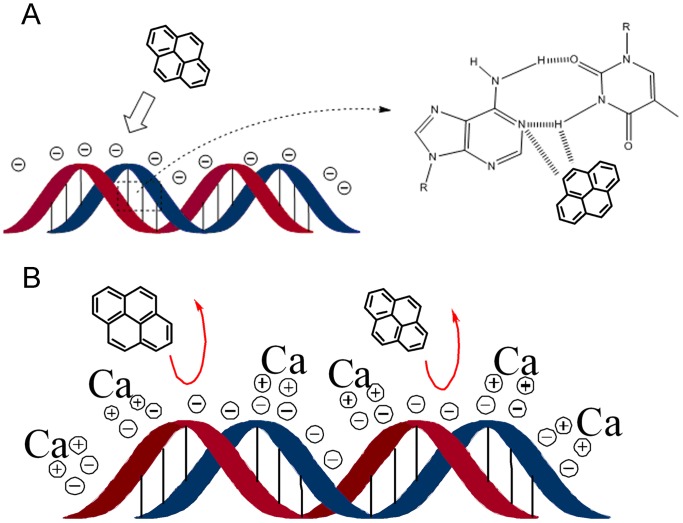
Diagram of the mechanism of Ca^2+^-inhibited PAH adsorption via blocking of the binding site in plasmid DNA. A hydrophobic interaction between pyrene and DNA bases occurred via a phosphate backbone structure (A). Ca^2+^-inhibition of the interaction between pyrene and DNA occurred via the capture of PAHs by DNA, thereby blocking the phosphate backbone structure of the DNA periphery (B).

The DNA double helix was stabilized primarily by two forces: hydrogen bonds between nucleotides and base-stacking interactions among the aromatic nucleobases [26]. Since hydrogen bonds were not covalent, they could be broken relatively easily and then rejoined. Aromatic and planar molecules known as intercalators fitted into the space between two adjacent base pairs. For an intercalator to insert between base pairs, the bases must separate, distorting the DNA strands by unwinding the double helix [24], [27], [28]. This inhibited both transcription and DNA replication, possibly causing a loss of gene expression (detailed mechanism presented in Figures 3, 4, 5, 6, 7, 8).

As shown in Figure 1, the transformation of plasmid DNA as a function of phenanthrene/pyrene concentration could be inhibited by planar PAH molecules. As phenanthrene/pyrene concentrations increased to 50 µg·L^–1^, the transformation efficiency of plasmid DNA (logarithm transformation, log_10_) decreased substantially. For phenanthrene, the transformation efficiency decreased from 4.67 to 3.2 log units as phenanthrene increased to 6.1 µg·L^–1^. The transformation efficiency then slightly increased to 3.62 log units as the phenanthrene concentration increased from 6.1 to 50 µg·L^–1^. Similarly, as shown in Figure 1, the presence of pyrene (<50 µg·L^–1^) also clearly inhibited DNA transformation. Transformation efficiency decreased from 4.67 to 3.36 log units as the pyrene concentration increased from 0 to 5 µg·L^–1^ and then increased slightly to a relatively steady state (3.44 log units) when the concentration of pyrene reached 50 µg·L^–1^. As well as the DNA transformation affected by phenanthrene, the slight increase in DNA transformation efficiency at higher pyrene concentrations might be related to the increasing enzyme resistance of DNA due to the association of DNA with PAHs [25]. However, the transformation efficiency of DNA in the presence of phenanthrene/pyrene was always lower than transformation in the absence of phenanthrene/pyrene, which suggested that PAH exposure was unfavorable for the DNA transformation.

### Ca^2+^-enhanced Low Transformation Efficiency of DNA Exposed to PAHs

Although the tested PAHs (phenanthrene/pyrene) inhibited DNA transformation, the presence of Ca^2+^ enhanced the low transformation efficiency of plasmid DNA exposed to phenanthrene/pyrene (Figure 2). In the absence of phenanthrene/pyrene, the transformation efficiency did not change with increasing Ca^2+^ concentration (0–50 mg·L^–1^). The transformation efficiency of plasmid DNA exposed to only Ca^2+^ always remained around 4.71 log units. These results suggested that the transformation efficiency of DNA could not be affected by solely increasing Ca^2+^ (to concentrations <50 mg·L^–1^).

Previous research has suggested that Ca^2+^ concentrations exceeding 80 mmol·L^–1^ (to concentrations >320 mg·L^–1^) promoted the transformation efficiency of plasmid DNA [1]. This mechanism was explained by the hydroxyl-calcium phosphate complex in DNA, which protected DNA against extracellular enzymatic degradation, facilitating DNA movement into the cell [17]. In the present study, low Ca^2+^ concentrations (0–50 mg·L^–1^) were not capable of facilitating DNA transformation, likely due to insufficient hydroxyl-calcium phosphate complex levels.

DNA transformation was inhibited at phenanthrene concentrations of only 2 µg·L^–1^ (Figure 2). Transformation efficiency was recovered by increasing Ca^2+^ concentrations from 0 to 50 µg·L^–1^. Maximum DNA transformation corresponded to 20 µg·L^–1^ of phenanthrene (Figure 3). At 20 µg·L^–1^ of phenanthrene only, the DNA transformation efficiency was 3.2 log units. When the Ca^2+^ increased to 10, 20, and 50 mg·L^–1^, the transformation efficiency recovered to 4.1, 4.3, and 4.28 log units, respectively. Similarly, at 0, 30, 40, and 50 µg·L^–1^ of phenanthrene, the transformation efficiency was also recovered by the addition of 0–50 mg·L^–1^ of Ca^2+^. This suggests that Ca^2+^ has a positive effect on the recovery of the transformation efficiency of plasmid DNA exposed to PAH contaminants.

In the presence of pyrene, Ca^2+^ also recovered rates of the low transformation efficiencies of plasmid DNA. In the absence of Ca^2+^, the transformation efficiencies at pyrene concentrations of 0, 2, 5, 10, 20, 30, and 50 µg·L^–1^ were 4.81, 3.38, 3.36, 3.50, 3.65, 3.51, and 3.45 log units, respectively. In contrast, as the Ca^2+^ concentration increased to 20 µg·L^–1^, the corresponding transformation efficiencies increased to 5.00, 3.95, 4.68, 4.82, 4.98, 5.02, and 5.05 log units, respectively. This suggested that although pyrene exposure weakened DNA transformations, the addition of Ca^2+^ clearly protected DNA against damage from pyrene and facilitated plasmid DNA recovery from the low transformation efficiencies caused by pyrene exposure.

### Ca^2+^ Control Over PAH-exposed Plasmid DNA Transformations

It was found that Ca^2+^ could impede the adsorption of both phenanthrene and pyrene by DNA and that the interactions between DNA and phenanthrene/pyrene were controlled by the addition of Ca^2+^. In the absence of Ca^2+^, DNA was found to combine with a greater quantity of phenanthrene/pyrene, which caused damage to the DNA and resulted in the low transformation efficiency of DNA exposed to PAHs. However, the addition of Ca^2+^ impeded the combination of DNA with phenanthrene/pyrene and therefore enhanced the transformation of DNA exposed to these contaminants.

As shown in Figure 3, the co-adsorption of Ca^2+^ and phenanthrene/pyrene by DNA provided an explanation for why the addition of Ca^2+^ facilitated recovery from the low transformation efficiency of plasmid DNA exposed to PAH contaminants. As shown in Figure 3, it was observed a distinct competitive adsorption between phenanthrene/pyrene and Ca^2+^. In absence of Ca^2+^, a linear correlation was observed between adsorbed phenanthrene and pyrene in solution. These results were in agreement with previous reports [29]–[32] and indicated that the partitioning of phenanthrene between DNA and the aqueous solution dominated the adsorptive process. In other words, DNA still had a large adsorptive capacity for phenanthrene when more adsorbate was added to the isothermal system [32]. However, when 3, 6, and 11 mg·L^–1^ of Ca^2+^ were added to the isothermal system, the amount of phenanthrene adsorbed by DNA gradually decreased compared to adsorption in the absence of Ca^2+^, and the shape of the adsorption curve suggested that the adsorption of phenanthrene by DNA trends toward saturation [30], [32]. This result indicated an inhibition of Ca^2+^ on the adsorption of phenanthrene by DNA.

Figure 3 illustrated the co-adsorption of Ca^2+^ and pyrene by DNA. The curvilinear pyrene adsorption curve sloped downward as Ca^2+^ increased from 0 to 3 mg·L^−1^, while the amount of pyrene adsorbed by DNA decreased. When 1 mg·L^−1^ of Ca^2+^ and more than 20 µg·L^−1^ of pyrene were added to the isothermal system, the curves for pyrene adsorption approached a state of equilibrium, suggesting that Ca^2+^ impedes the adsorption of pyrene by DNA.

### Interaction of DNA, PAHs, and Ca^2+^ Based on Different Bonding Models

Small differences in the FTIR spectra demonstrated that phenanthrene/pyrene might affect DNA base pairs (Figure 4). Based on the data in Table 1, the absorption band near 1699 cm^–1^ was attributable to adenine. Following the reaction with phenanthrene/pyrene (Figure 4B and C), the absorption band of DNA–phenanthrene was enhanced at 1699 cm^–1^, whereas the absorption band of pyrene near 1699 cm^–1^ was weaker than that for pure DNA. In addition, the absorption band of adenine also became weak for the DNA–Ca^2+^-phenanthrene and DNA–Ca^2+^-pyrene treatments (Figure 3D and E). This suggested that phenanthrene and pyrene react with DNA via interactions between hydrophobic adenine and hydrophobic PAHs.

**Table 1:** The absorption bands of DNA from FTIR spectra and their corresponding functional groups.

Adsorption bands (cm^−1^)	Functional groups	References
<962	Symmetrical stretch vibration of PO_2_^−^	[33]
970–950	DNA backbone	[21], [34]
1020–1010	Furanose vibration	[35], [36]
1053	Stretch vibration of P-O or C-O	[37], [38]
1080	Symmetrical stretch vibration of phosphate functional groups (PO_2_^–^O-CH_2_)	[7], [34]
1236	Dissymmetrical stretch vibration of PO_2_^−^	[34]
1369	Guanine	[34], [39]
1420 and 1488	DNA structure	[37],
1531	Imidazole ring	[43], [44]
1651	Stretch of C = C or C = N in base	[43], [44]
1699	Bands of protonated adenine	[43], [45]

Some studies have suggested that PAHs might influence the bases and structure of DNA [22]. Table 1 suggested that the protonation/deprotonation of adenine might be linked to the banding site located at 1699 cm^–1^. However, hydrophobic PAHs could not provide a free proton for adenine due to their molecular conformation. Therefore, the weak interactions between DNA and phenanthrene/pyrene, based on molecular forces, might dominate alteration of the absorption band for this binding site. Polar DNA molecules might induce a relative displacement between the electron cloud and the atomic nucleus of nonpolar PAHs, causing those dipoles in PAH molecules with excellent induction forces to be attracted to the innate dipole in polar DNA molecules. This interaction might induce a change between the protonation and deprotonation of DNA adenine, causing a stronger or weaker FTIR absorption, respectively, of adenine.

The DNA band near 1080 cm^–1^ is linked to the symmetrical stretching vibration of phosphate functional groups (PO_2_^–^–O–CH_2_) and was absent from either the DNA–phenanthrene-Ca and DNA–pyrene-Ca treatment (Figure 4D and E), but presented in both DNA–phenanthrene and DNA–pyrene. Therefore, we suspected a strong association between PO_2_^–^ and Ca^2+^. Such an association could be related to the production of hydroxy calcium phosphate. In other words, the ionization of hydrogen in the “–POOH–” groups of DNA led to the formation of electronegative phosphate functional groups “–POO^–^–” that captured Ca^2+^ (Figure 5). Because of differences in charge between –POO^–^– groups and Ca^2+^, each Ca^2+^ would in theory combine with two –POO^–^–, and consequently, a chain of –POO^–^– groups might lock up neighboring nucleotides. Such a chain also induced a slight change in DNA structure, as observed from the FTIR adsorption band near 962 cm^–1^ (Figure 4).

Results from the XPS (Figure 6) further confirmed the above-mentioned hypothesis regarding the formation of electrovalent bonds between the phosphate groups of DNA and Ca^2+^. The binding energy (eV) of oxygen for DNA (532 eV) increased to 535.3 eV for Ca^2+^–DNA, whereas no change was observed in the binding energy of oxygen for DNA–pyrene when compared to DNA. This showed the formation of strong electrovalent bonds between phosphate and Ca^2+^.

To exclude possible DNA–PAH adducts and to further confirm the physical interaction between phenanthrene/pyrene and DNA, the nucleotides after phenanthrene/pyrene–DNA interactions were analyzed using HPLC-MS. Adductive combinations of PAHs and DNA are based on a firm covalent bond. If the interaction between PAHs and DNA was based on this covalent bond, then the adducts of PAHs–DNA should be detectable using MS. Figure 7 shows chromatogram peaks for four bases (cytosine, guanine, adenine, and thymine) with their corresponding retention times. With the exception of the four DNA base peaks, no new MS peaks were observed from the chromatograms of bases for DNA–phenanthrene, DNA–phenanthrene-Ca, DNA–pyrene, or DNA–pyrene-Ca treatments. This indicated that pure DNA did not combine with PAHs through the usual covalent bond, and that the interaction between DNA and PAHs was based on a weak molecular force. This result is also supported by the above FTIR.

Therefore, we believed that the interactions between DNA and PAHs were related to weak molecular forces, and that interactions between DNA and Ca^2+^ were mediated by an electrovalent bond. The formation of an electrovalent bond between DNA and Ca^2+^ weakened the interaction between DNA–PAHs, decreasing the effect of PAHs on hydrogen bonds in DNA strands, thereby promoting the low transformation efficiency of DNA.

We further proposed the mechanism of Ca^2+^-enhancement of transformation efficiency shown in Figure 8. The Ca^2+^-enhanced low transformation efficiency of plasmid DNA exposed to PAH contaminants might be related to the interactions among DNA, PAHs, and Ca^2+^ based on different types of chemical bonds. Initially, the –POO^–^– groups of DNA combine with Ca^2+^ via electrovalent bonding, altering DNA’s superficial properties and reducing its surface electrical charges. The polarization of PAH molecules with an unsaturated π electron by Ca–DNA was then inhibited by a relative displacement between the electron cloud and the nucleus, such that dipole formation did not occur. The end result was that PAH molecules were not adsorbed onto the Ca–DNA complex. Consequently, the effect of PAH contamination was weakened by Ca^2+^ on the surface of DNA, resulting in an enhanced transformation efficiency.

In conclusion, damage to plasmid DNA by PAHs was generally considered to be the main inhibitory mechanism underlying the low transformation efficiency of DNA during HGT. Furthermore, Ca^2+^ might lessen the inhibitory effect of PAHs on plasmid DNA. FTIR spectroscopy and MS analyses revealed a hydroxyl calcium phosphate related to an electrovalent bond between DNA and Ca^2+^ and a lack of covalent bonds in “DNA–PAH” adducts. Therefore, the low transformation efficiency of DNA was attributable to the breaking of a hydrogen bond by planar PAH molecules. Strong electrovalent bonds were formed between Ca^2+^ and –POO^–^– groups in DNA, weakening the interaction between PAHs and DNA, reducing the damage to hydrogen bonds in DNA by PAHs by isolating DNA molecules from PAHs, and consequently enhancing transformation efficiency. These findings would strengthen our understanding of how anthropogenic trace PAHs in natural environments affected biological heredity and variation, ecological and genetic diversity, and biological evolution.
